# Upwelling modulation of functional traits of a dominant planktonic grazer during “warm-acid” El Niño 2015 in a year-round upwelling area of Humboldt Current

**DOI:** 10.1371/journal.pone.0209823

**Published:** 2019-01-14

**Authors:** Victor M. Aguilera, Ruben Escribano, Cristian A. Vargas, M. Teresa González

**Affiliations:** 1 Centro de Estudios Avanzados en Zonas Áridas (CEAZA), Coquimbo, Chile; 2 Facultad de Ciencias del Mar, Depto. Biología Marina, Universidad Católica del Norte, Coquimbo, Chile; 3 Instituto Milenio de Oceanografía and Departamento de Oceanografía, Facultad de Ciencias Naturales y Oceanográficas, Universidad de Concepción, Concepción, Chile; 4 Aquatic Ecosystem Functioning Lab (LAFE), Department of Aquatic Systems, Faculty of Environmental Sciences and Environmental Sciences Center EULA Chile, Universidad de Concepción, Concepción, Chile; 5 Center for the Study of Multiple-drivers on Marine Socio-Ecological Systems (MUSELS), Universidad de Concepción, Concepción, Chile; 6 Instituto de Ciencias Naturales Alexander von Humboldt, Universidad de Antofagasta, Antofagasta, Chile; Stazione Zoologica Anton Dohrn, ITALY

## Abstract

Climate change is expected to exacerbate upwelling intensity and natural acidification in Eastern Boundaries Upwelling Systems (EBUS). Conducted between January-September 2015 in a nearshore site of the northern Humboldt Current System directly exposed to year-round upwelling episodes, this study was aimed at assessing the relationship between upwelling mediated pH-changes and functional traits of the numerically dominant planktonic copepod-grazer *Acartia tonsa* (Copepoda). Environmental temperature, salinity, oxygen, pH, alkalinity, chlorophyll-*a* (Chl), copepod adult size, egg production (EP), and egg size and growth were assessed through 28 random oceanographic surveys. Agglomerative clustering and multidimensional scaling identified three main di-similitude nodes within temporal variability of abiotic and biotic variables: A) “upwelling”, B) “non-upwelling”, and C) “warm-acid” conditions. Nodes A and B represented typical features within the upwelling phenology, characterized by the transition from low temperature, oxygen, pH and Chl during upwelling to higher levels during non-upwelling conditions. However, well-oxygenated, saline and “warm-acid” node C seemed to be atypical for local climatology, suggesting the occurrence of a low frequency oceanographic perturbation. Multivariate (LDA and ANCOVA) analyses revealed upwelling through temperature, oxygen and pH were the main factors affecting variations in adult size and EP, and highlighted growth rates were significantly lower under node C. Likely buffering upwelling pH-reductions, phytoplankton biomass maintained copepod reproduction despite prevailing low temperature, oxygen and pH levels in the upwelling setting. Helping to better explain why this species is among the most recurrent ones in these variable yet productive upwelling areas, current findings also provide opportune cues on plankton responses under warm-acid conditions, which are expected to occur in productive EBUS as a consequence of climate perturbations.

## Introduction

A comprehensive understanding of oceanographic processes controlling spatial [1–3] and temporal [3,4,5] variations in pH and *p*CO_2_ conditions in coastal regions have improved our capacity to identify natural and anthropogenic signals and their future trends [6,7,8]. The uptake of anthropogenic CO_2_ has caused low pH, high *p*CO_2_ waters to shoal since preindustrial times, and in consequence are now part of the waters currently being upwelled in some coastal regions of productive Eastern Boundary Upwelling Systems (EBUS) [2,8]. While there seems to be consensus regarding the trend of increased upwelling intensity in the future due to intensification of upwelling favorable winds [9] with consequences in ocean acidification (OA) [9,10], less attention has been devoted to understanding the impact of discrete low pH-events (“event effects”) [11]. The magnitude and frequency of these events are also increasing due to climate change and they have been recognized as introducing important effects on the structure of communities and ecosystem functioning [12].

Upwelling areas are characterized by a high temporal variability, from hours to months, in consequence, upwelling driven pH-changes might constitute a significant environmental factor affecting short-term but ecologically functional plankton processes, such as growth and reproduction [13,14,15]. In the plankton system of these productive upwelling zones, short life-cycle (e.g. weeks to months) copepods are very successful in coastal areas [16], often becoming the bulk of zooplankton biomass [14,17]. However, the role of pH as an environmental regulator of copepod survival [18,19,20], development [19], feeding [21,22], and reproduction [23,24,25] appears controversial in pelagic species. Importantly, natural variability of the species’ habitat has been scarcely considered in the design of this relatively large and growing literature body of experimental studies [26,27,28]. The omission of the environmental history can lead to uncertain interpretations of copepod physiology under pH-variations, which may not necessarily reflect current local environmental-biological coupling or future responses to global stressors [29].

In the Humboldt Current System (HCS), one the most productive EBUS [30], the neritic copepod *Acartia tonsa* has consistently been found among the most abundant and prevalent species [13,14,17]. Inhabiting temperate waters in nearshore environments between 20 and 30° S off the Chilean coast, *A*. *tonsa* is a small-sized species that efficiently and rapidly converts food into eggs [14,15,17]. Yet, their reproduction and population recruitment are thought to occur permanently in these areas [31,32]. Prevalence of the *A*. *tonsa* abundance in upwelling areas can be favored by their life history traits, which might be unaffected by upwelling pH-changes. In this study, we aimed at testing this hypothesis by assessing environmental variability (temperature, salinity, oxygen, pH, alkalinity and chlorophyll) and copepod traits (body size, egg reproduction, egg size, and growth) in the year-round upwelling center off Antofagasta (23° S) during the year 2015. Di-similitude in the abiotic and biotic matrices observed during 28 oceanographic surveys allowed us to identify two nodes of temporal variability which fit well within the upwelling seascape phenology (i.e., upwelling and non-upwelling conditions). In addition, a third “warm-acid” node suggested the influence of a remotely originated oceanographic perturbation upon which, temperature, oxygen and pH strongly impacted copepods’ body size and reproduction.

## Methods

### Ethic statements

Field surveys and animal collection was under the agreement of Chilean Hydrographic Service of the Chilean Navy (SHOA ordinario 30270/24/466).

### Study area

**Fig 1:**
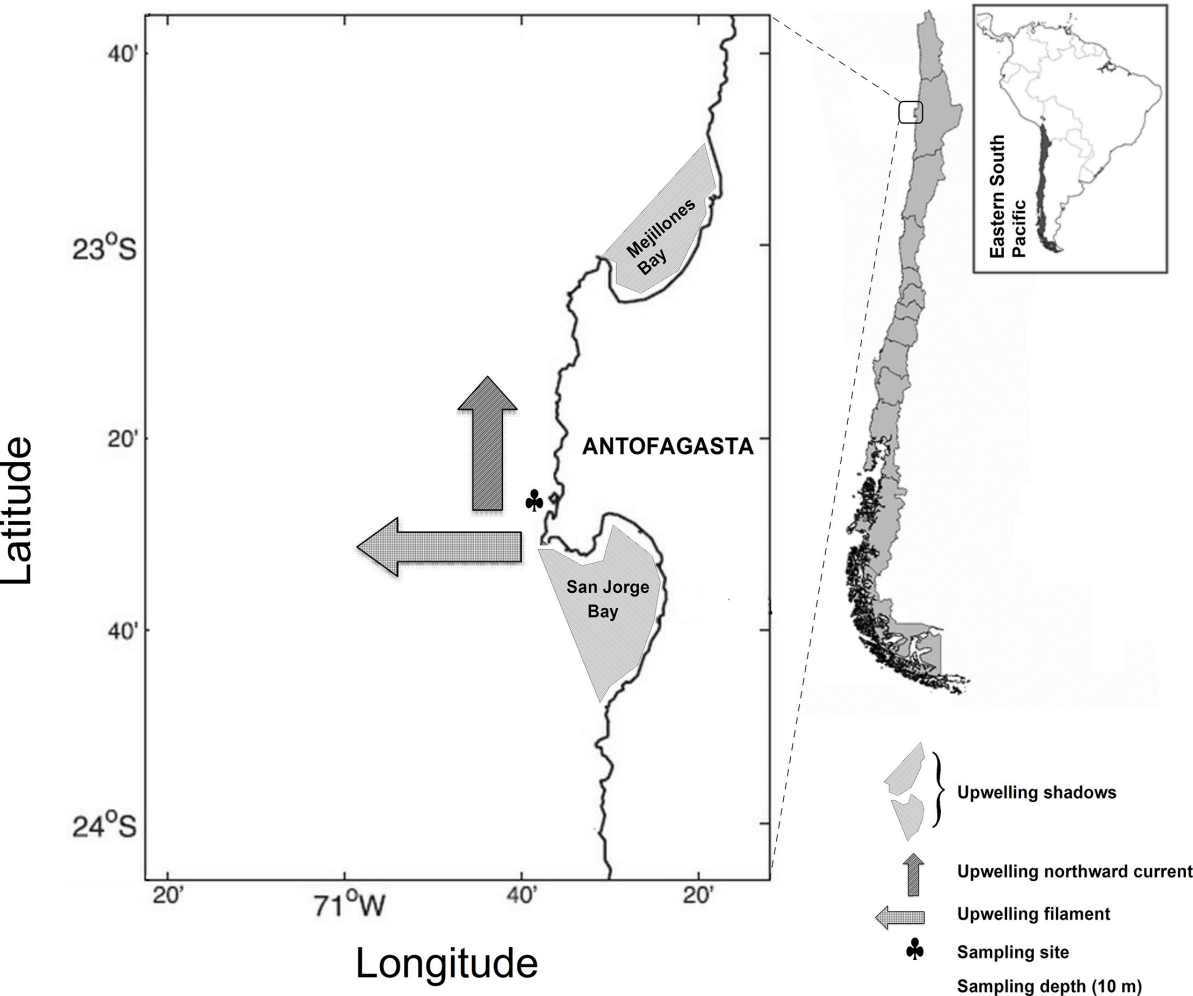
Map of the study area. Monitoring was carried out during 2015 in a coastal site exposed directly to upwelling filaments. The former generates nearshore northward currents, which later enter into northern Mejillones Bay as upwelling shadows.

Upwelling variability was characterized through 28 random oceanographic campaigns in the upwelling area off Antofagasta (S 23°27’23, W 70°37’13) of the HCS (Fig 1). Sampling was performed during morning time (10:00 and 11:30 am) from January to September 2015 in nearshore waters exposed directly to upwelling filaments and meandering currents [33]. At this latitude, coastal dynamics takes place over a very narrow continental shelf (< 20 km) and it responds closely to equatorward upwelling favorable winds prevailing year-round [34]. Freshly upwelled waters in advective open areas are characterized by high nutrients and *p*CO_2_ conditions, but low oxygen and pH levels [1,35]. After upwelled, a portion of these also colder waters intrude into northern nearby Mejillones Bay conforming what is known as an upwelling “shadow”, becoming warm and stratified, and so giving rise to substantial spatial gradients in the ambient suitability for planktonic components [36,37].

### Oceanographic environmental conditions and animal collection

Environmental conditions were assessed on a costal station (1 km from the coast) by measuring temperature, salinity, oxygen, seawater pH, total alkalinity, and chlorophyll-*a* (Chl) concentration (Fig 1, Table 1). Temperature, salinity and dissolved oxygen profiles were recorded from above the bottom (∼ 40 m) to the surface through vertical deployments of a calibrated SeaBird SBE19 plus CTD, equipped with a Wet Star oxygen sensor. Up to 30 L of seawater were obtained with a 10 L Niskin bottle at 10 m depth (Table 1) for Chl, pH and total alkalinity measurements. This depth was chosen because it is thought to represent the actual habitat of the dominant neritic copepod species in this area [32,38]. Size-fractioned Chl (Chl_> 20 and Chl_< 20 μm) concentration was analyzed in triplicate and measured by using a TD Turner fluorometer; after that 200 mL subsamples were sieved with a 20 μm mesh sieve and filtered onto a GF/F filter. Chl was extracted in the dark for 24 h in 90% acetone v/v [39]. Seawater pH_@25°C_ was measured triplicate within 1 h from time of collection in a closed 25 mL cell thermostated using a Metrohm 827 pH meter (input resistance, > 1×10^12^ Ohm, 0.1 mV sensitivity and nominal resolution at 0.01 pH units) and a glass combined double junction Ag/AgCl electrode (Aquatrode PT1000, N/P 6.0257.000) calibrated with 4, 7 and 8 Tris buffers. Samples for total alkalinity analysis were collected in borosilicate glass bottles with ground glass stoppers (250 mL) and poisoned with 10 μL HgCl_2_. Total alkalinity was determined using the open-cell titration method [40], by means of an automatic Alkalinity Tritrator AS-ALK2 Apollo SciTech. All samples were analyzed at 25° C (± 0.1° C) with thermal regulation using a water-bath. Alkalinity, temperature, salinity, and pH_@25°C_ were used to calculate *in situ* pH (and other parameters of the carbonate marine system) through the program CO_2_SYS version 01.05 [41].

**Table 1:** Details of measurements and estimations conducted to assess oceanographic and biological variability in the upwelling setting.

Year	Water column depth (m)	Measurements (*) Estimations (+)	Sampling depth (m)	Monitoring period
2015	40	Temperature *	10	Jan. to Sept.
		Salinity *	10	Jan. to Sept.
		Dissolved oxygen *	10	Jan. to Sept.
		pH *	10	Jan. to Sept.
		Alkalinity *	10	Jan. to Sept.
		Chlorophyll-*a* ^*+*^	10	Feb. to Sept.
		Body size *	20–10	Jan. to Sept.
		Egg production ^+^	20–10	Jan. to Sept.
		Egg size *	20–10	Jan. to Sept.
		Growth rate ^+^	20–10	Jan. to Sept.

Plankton samples were collected simultaneously with oceanographic surveys characterizing hydrographic conditions using a 200 μm WP2 plankton-net equipped with a 1 L non-filtering cod-end, which was hauled vertically from ~20 to 10 m depth (Table 1). Within 2 h of collection, undamaged, mature, and visibly healthy females of *A*. *tonsa* were sorted under a Leica EZ4HD stereomicroscope, transferred individually to 300 mL beakers and stored at the same temperature observed at 10 m depth (14–16°C) during sampling until setting up the experiments. Temperature was adjusted in a cold room whose intra-inter daily thermal variations were ≤ 0.4°C. From the copepod samples, up to 40 females were preserved immediately in 90% ethanol for body length (BL) determinations. BL was expressed as body mass by means of the length-weight regression reported by Uye [42] and converted to body carbon assuming a specific-carbon content of 45% [43] to estimate later growth rates (see below).

### Field egg production

For estimates of egg production rates (EP) groups of 25–30 *A*. *tonsa* females were gently pipetted individually into 300 mL acid-washed crystallizing dishes loaded with filtered (0.2 μm) seawater without food. Females were incubated at *in situ* temperature and EP (egg fem^-1^ d^-1^) was estimated as the number of eggs produced over 24 h [15]. Some of these eggs (20–30) were preserved immediately in 90% ethanol to estimate the egg size using an inverted microscope Olympus IX-51. Weight-specific growth rates (d^-1^) were assumed to be in linear form with fecundity, as eggs are shed and not added to female body carbon, such that egg outputs represented total growth of the adult female according to the equation of Hirst and Lampitt [44]:

g=(We/Wa)(24/t)

*We* is the egg carbon quantity produced over time (*t* = days) and *Wa = body carbon*. Accounting daily pulses of egg production [45], we assumed female body carbon was steady state between spawning.

### Data analysis

#### Pre-processing

Data was evaluated to detect outliers by applying a Grubbs Test, which detected outliers in pH, Chl_<20 and growth (p = 0.0001). Collinearity among factors was explored by means of both principal component analysis (PCA) and Durbin Watson test. Alkalinity was collinear with salinity and thus, alkalinity was not considered in further analysis. Normality was assessed by means of Lilliefors (p <0.01) and Shapiro–Wilk’s W tests, while homoscedasticity (Cochrane and Hartley test) showed variance among predictor factors was homogeneous.

#### Data processing

To look for relevant features within the temporal coverage of environmental and biological variability, distance matrices were made considering Julian day as categorical predictor and ambient factors (Euclidean) and copepods responses (Bray-Curtis) as variability features. Data were normalized, one attribute per value by standard deviation, from -1 to 1, and continuized by using linear correlation (Spearman rank) between the rank of the values, remapped as a distance in a (0, 1) interval. Then, a hierarchical agglomerative clustering was constructed utilizing a weighted function linkage. Di-similitude within sampling days was thus grouped in three top temporal nodes capturing ≥30% of variability. Additionally, a multi-dimensional scaling (MDS) test, which is a low-dimensional projection of better possible fit between distances of two points, was applied to validate clustering outputs. Differences among temporal nodes (i.e. classes) provided by the hierarchical clustering (and MDS) were assessed by means of a one-way ANOVA test, considering temporal nodes as categorical predictors and oceanographic factors and copepods responses as continuous variables. The relationship among environmental factors was assessed by means of a distance map supported by Spearman rank correlations, whereas the relationship between ambient variability and copepod responses was assessed through a multivariate linear discriminant analysis (LDA), and covariance analysis (ANCOVA), after cos-normalization of data. Both LDA and ANCOVA operate without restrictions of normal distribution requirements and while LDA aims at explain a categorical variable by continuous independent variables, ANCOVA is similar than ANOVA but without considering the predictive effect due to linear regressions among co-variables. Data pre-processing and graphics were performed in Orange package version 3.14 [46] while statistical tests were performed in STATISTICA 10.

## Results

### Data quality

The accuracy for both alkalinity and pH determinations was controlled against certified reference material (A. Dickson, USA) [40]. Accordingly, uncertainties of alkalinity and pH estimates were 3 μmol kg^-1^ and 0.03 pH-units, respectively. Alkalinity values were not considered in analysis of temporal variations due to the fact that it was aliased with salinity. Moreover, pH uncertainty was not only lower than standard deviation for both the whole period (± 0.09 pH units), but also it was lower than the standard deviation observed among cluster nodes (±0.07 pH units). Therefore, uncertainties in pH measurements should not be expected to influence the comparison among clusters neither should the consideration of pH as a variability factor for copepod responses.

### Temporal variability of abiotic and biotic variables

**Fig 2:**
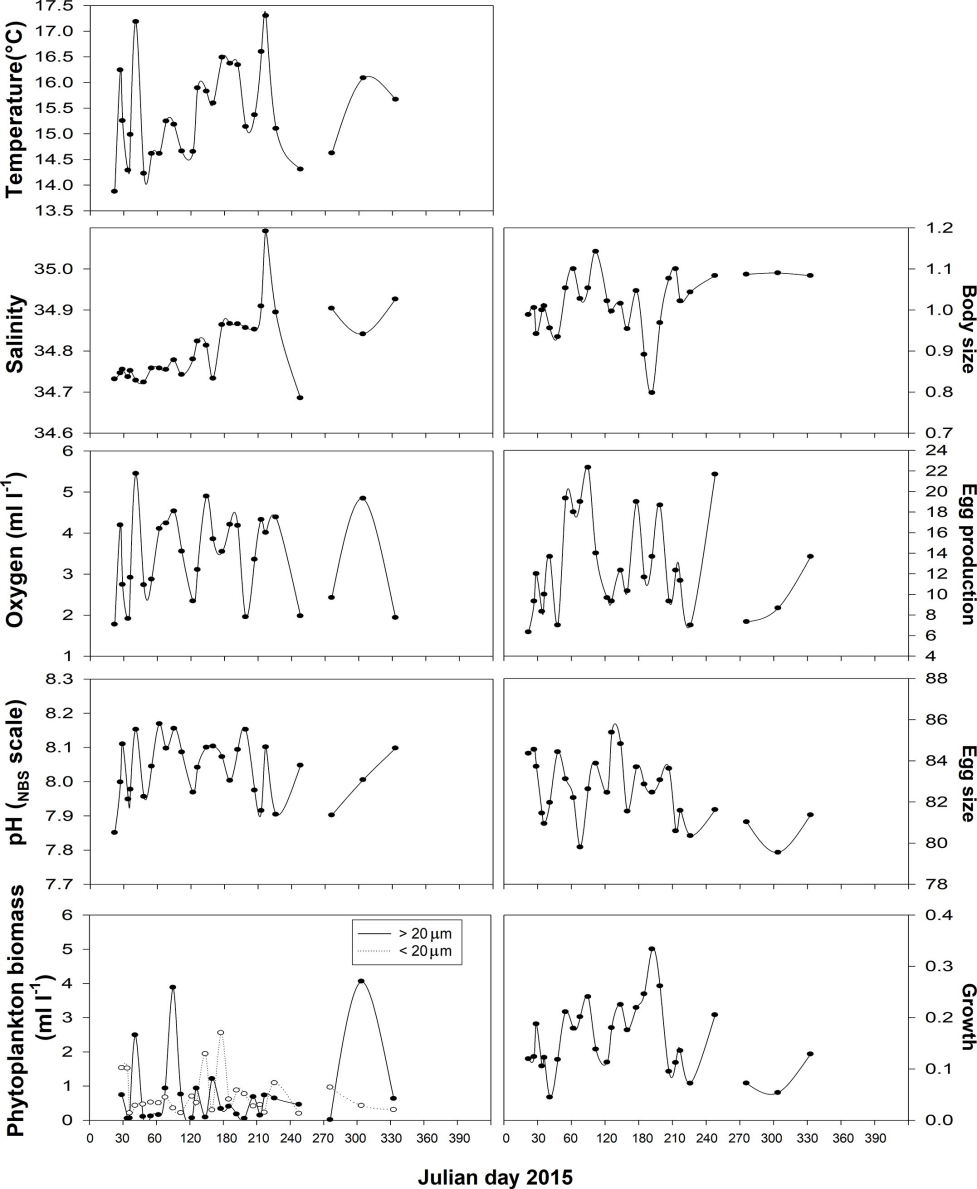
The abiotic and biotic temporal variability in the study area. Daily means values of oceanographic parameters and copepod responses after 24 random field surveys.

Graphic representations of temporal variability observed during the study in both abiotic and biotic variables are shown in Fig 2. Temperate-to-cold conditions (< 16°C) as well as relatively low oxygen (≥ 2 mL L^-1^) levels were observed during the first quarter of the study. During this period, salinity values, relatively high, varied in a very narrow range (34.7–34.8), but increased steadily up to 35.1 in the period immediately following. Seawater pH varied between 7.8 and 8.1 pH units, the mean value tended to be around 8.04 pH units although lower values occurred randomly during the study period (Fig 2). Upon this environmental background, relatively low phytoplankton biomasses (i.e. estimated as Chl concentration), were often observed in the sampling site, since mean values of Chl were < 2 μg L^-1^. With respect to copepod traits, adult size varied widely (0.7–1.4 mm) during the roughly eight months of study. Fecundity levels–estimated as egg production (EP)–were moderate (13±6 egg fem^-1^ d^-1^) during this study, varying between 2 and 24 egg fem^-1^ d^-1^. Egg size averaged 83±3 μm varying between 77 and 90 μm, whereas growth varied within one order of magnitude, from 0.01 to 0.4 d^-1^ (0.15± 0.08 d^-1^).

### Nodes of temporal variability

**Fig 3:**
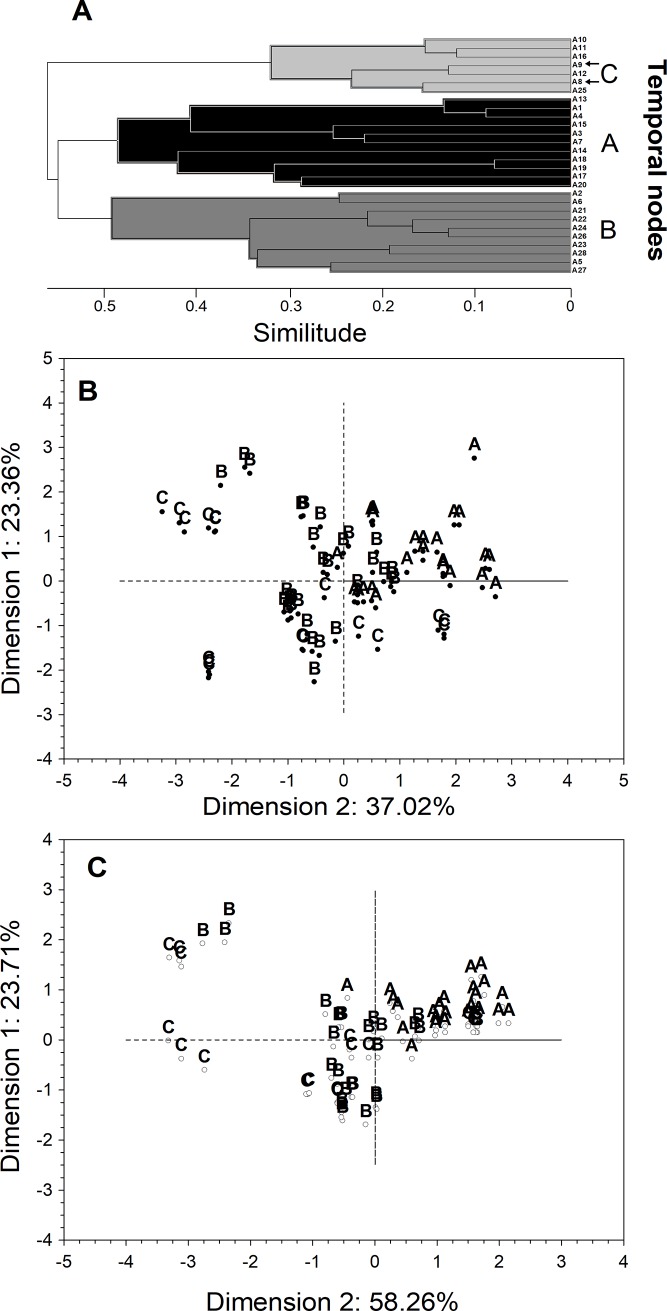
Three main nodes captured much of the observed abiotic and biotic temporal variability. Black arrows in hierarchical clustering (A) denote extremely warms days, which, if excluded from the MDS test (B), increased total explained variance by the temporal partitioning (C).

Supported by Euclidean distances (≥ 30%), the hierarchical agglomerative clustering identified three main nodes of variability: A, B, and C (Fig 3A). These findings were supported by a multi-dimensional scaling (MDS) test, whose first two dimensions explained 60.4% of the total temporal variance (Fig 3B), and showed cluster node C interrupted the random transitions from A to B (S1 File). By examining the sampling days grouped under each temporal node, most days grouped in node C belonged to the winter season with the exception of two very warm days observed in summer (see below). If these extremely but historical warm days are excluded from the MDS, total explained variance increased up to 81.2% (Fig 3C). Julian days, median ambient conditions and copepod traits grouped under each temporal node are listed in Table 2. Based on the former metrics, each node was assigned with a nominal condition within the upwelling seascape phenology.

**Table 2:** Summary of sampling days and median environmental conditions observed under each temporal node. Clustering segregation was supported by a multi-dimensional scaling (MDS) test.

Cluster node A				Cluster node B				Cluster node C			
Nominal condition				Nominal condition				Nominal condition			
“Upwelling”				“Non- upwelling”				“Warm-acid”			
Code	Julian day	Austral season	Median parameters	Code	Julian day	Austral season	Median parameters	Code	Julian day	Austral season	Median parameters
A1	22	Summer	Temp. = 14.8	A2	27	Summer	Temp. = 15.3	A10	75	Summer	Temp. = 16.1
A3	29	Summer	Sal. = 34.8	A5	36	Summer	Sal. = 34.8	A11	82	Winter	Sal. = 34.9
A4	34	Summer	Oxy. = 2.8	A6	41	Summer	Oxy. = 3.7	A16	118	Winter	Oxy. = 4.2
A5	36	Summer	pH = 8.01	A21	153	Fall	pH = 8.09	A9	68	Winter	pH = 7.99
A7	55	Summer		A22	157	Fall		A12	92	Winter	
A14	104	Summer		A23	166	Fall		A8	62	Winter	
A15	110	Fall		A24	188	Winter		A25	216		
A17	125	Fall		A26	244	Winter					
A18	132	Fall		A27	273	Winter					
A19	139	Winter		A28	344	Winter					
A20	147	Winter									

**Fig 4:**
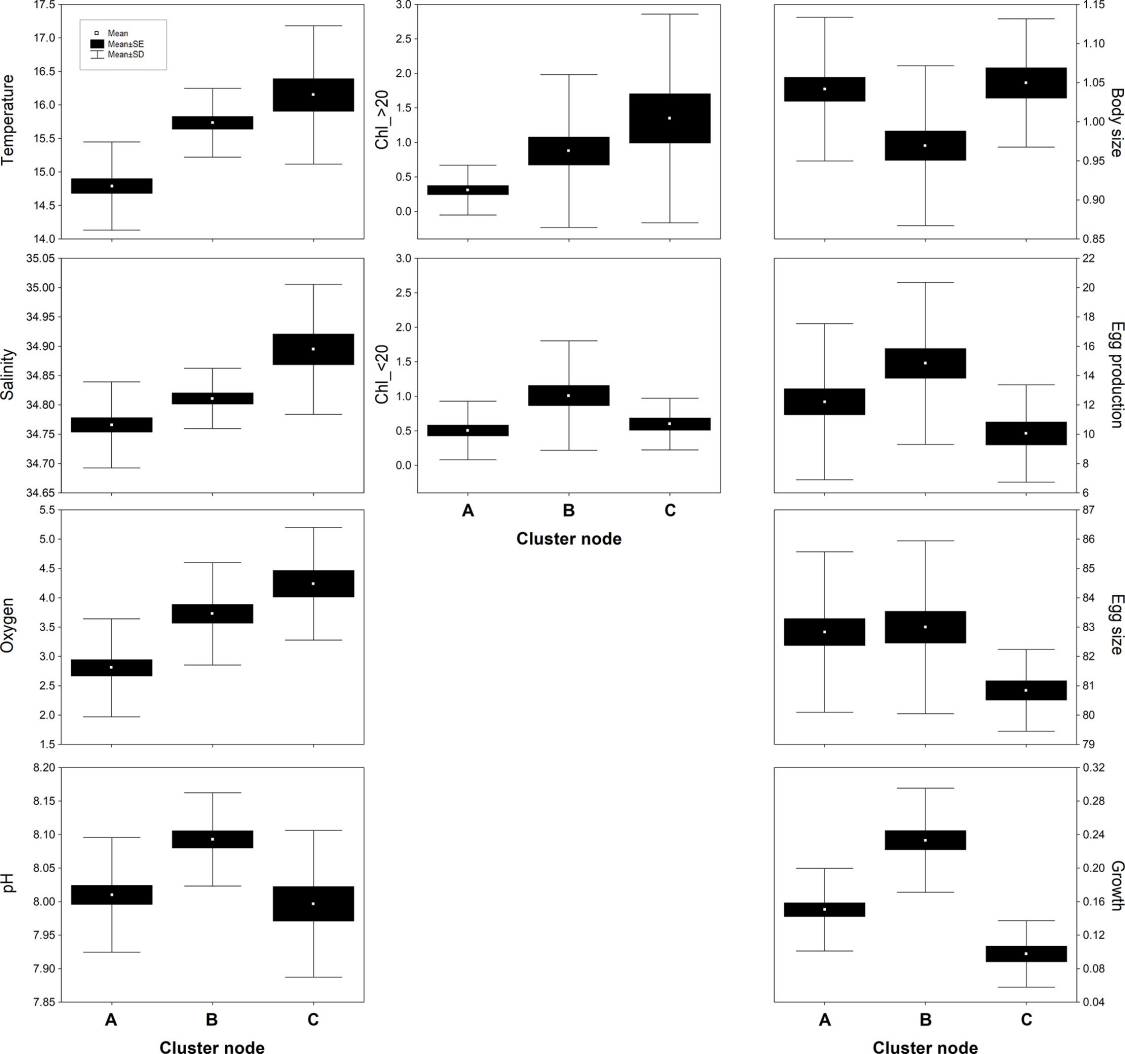
Mean values of abiotic and biotic variables observed under each temporal node. Box plots were computed after clustering of environmental factors and copepod responses: A (n = 11), B (n = 10), and C (n = 7).

Results of the statistical (one-way ANOVA/MANOVA test) comparison of environmental conditions and copepod responses observed under each cluster node are shown in Table 3. Significant differences among cluster nodes were observed in all abiotic and biotic parameters, although the trend of change was not uniform neither among environmental nor biological variables. The mean values, standard error and standard deviation of each parameter in relation to cluster nodes are shown as box plots in Fig 4. Accordingly, node A or “upwelling” was characterized by low temperature, oxygen, pH and high salinity levels, whereas higher values of the former parameters were observed in node B or “non-upwelling”. Both Chl_>20 and Chl_<20 almost followed the same pattern of change and tended to increase from A to B conditions. In contrast, no clear variability pattern was observed among copepod responses. For example, body size was higher under “upwelling” conditions contrary to growth, which was higher under “non-upwelling” conditions, whereas EP and egg size did not change among these nodes. Node C in turn, involved the highest temperature, oxygen, salinity and Chl_>20 levels, while pH and Chl_>20 returned to levels observed during “upwelling” conditions. This “warm-acid” node C also involved the lowest EP, egg size and growth levels, while body size did not change (Fig 4).

**Table 3:** Results of one-way ANOVA test applied to analyze variability in oceanographic factors and copepod responses with respect to cluster nodes. Fisher LSD post-hoc analysis denotes the trend of change among cluster nodes.

Source of variability	Variable	*d*.*f*.	*F*-value	*p*- value	Post-hoc
Temporal	Temperature	2, 84	33	0.001	A<B<C
node	Salinity	2, 84	19	0.001	A<B<C
	Oxygen	2, 84	26	0.001	A<B<C
	pH	2, 84	16	0.001	A<B>C
	Chl_>20	2, 78	10	0.01	A>B<c
	Chl_<20	2, 78	10	0.01	A = B<C
	Body size	2, 84	10	0.01	A = B>C
	Egg production	2, 84	11	0.003	A = B>C
	Egg size	2, 84	9.5	0.01	A<B = C
	Growth	2, 84	44	0.001	A<B<C

### Environmental and biological coupling

**Fig 5:**
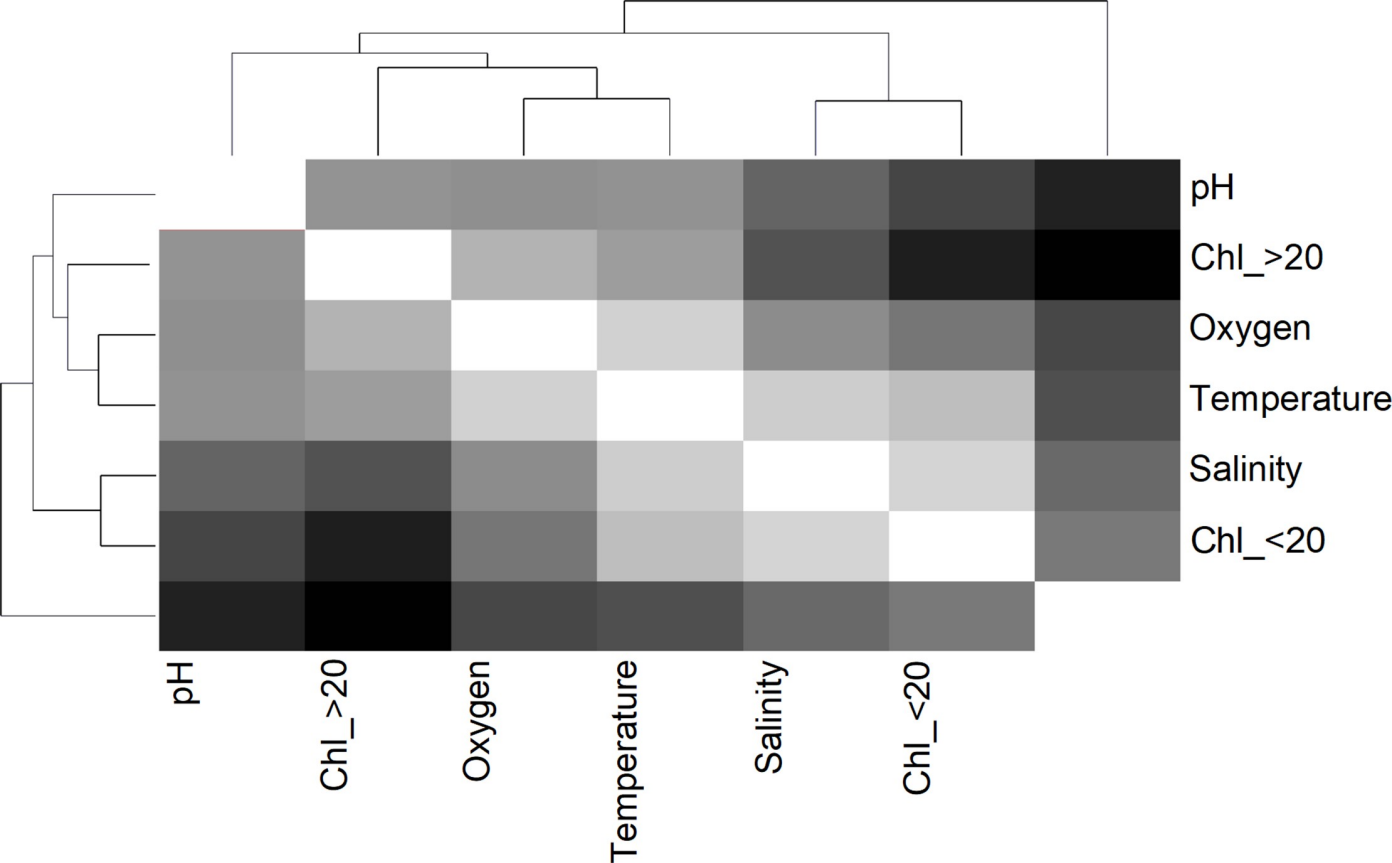
Map of significant correlations among oceanographic factors. Supported by Spearman rank correlations, the distance map suggests how the abiotic ambient changes from one temporal node to the other.

**Fig 6:**
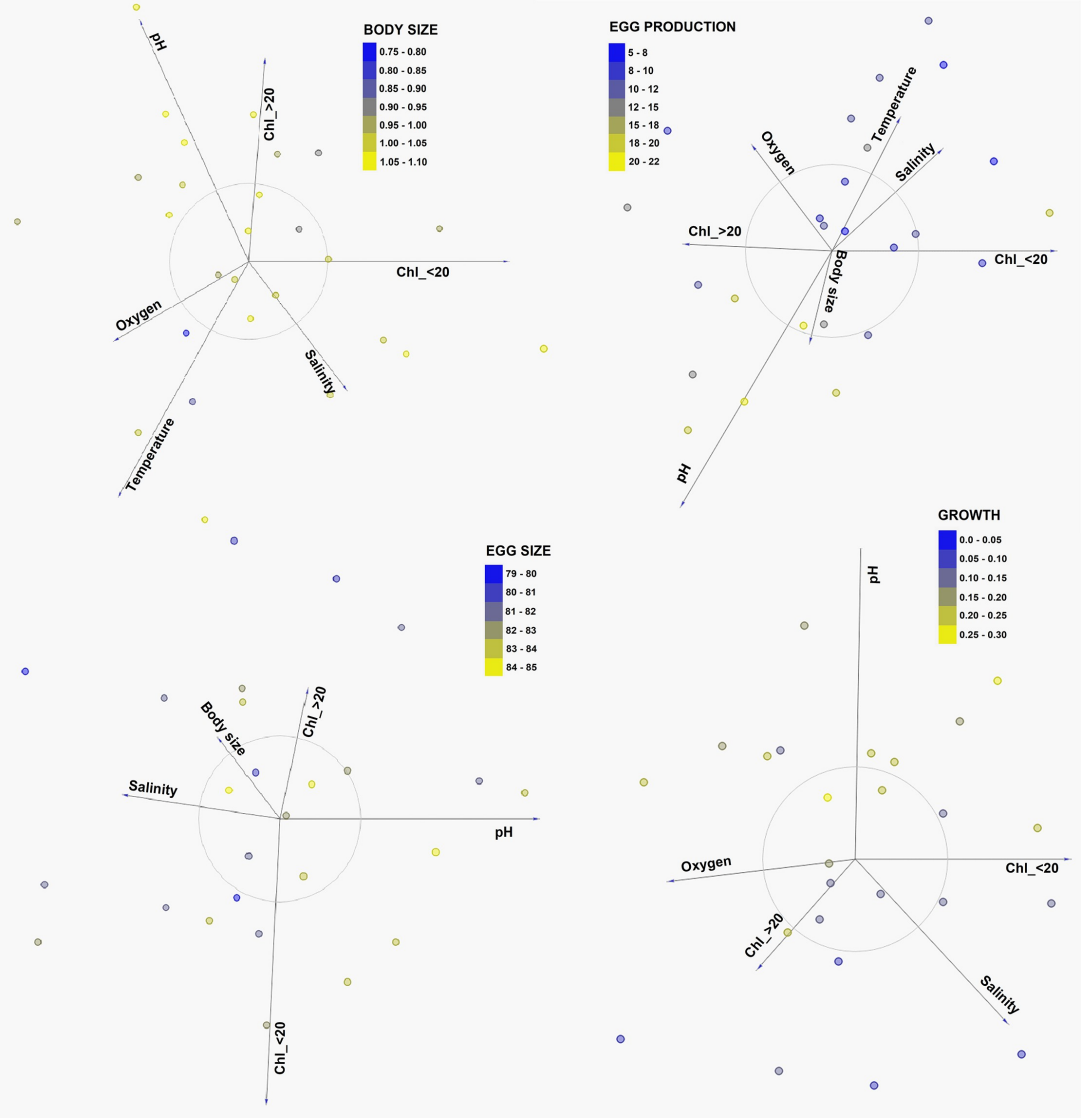
Different copepod responses modulated by the upwelling. Three-dimensional ordination of copepod traits (body size, egg production, egg size and growth) with regards to environmental factors after a LDA test was applied. Total explained was 61% (body size), 56% (egg production), 49% (egg size) and 60% (growth).

**Fig 7:**
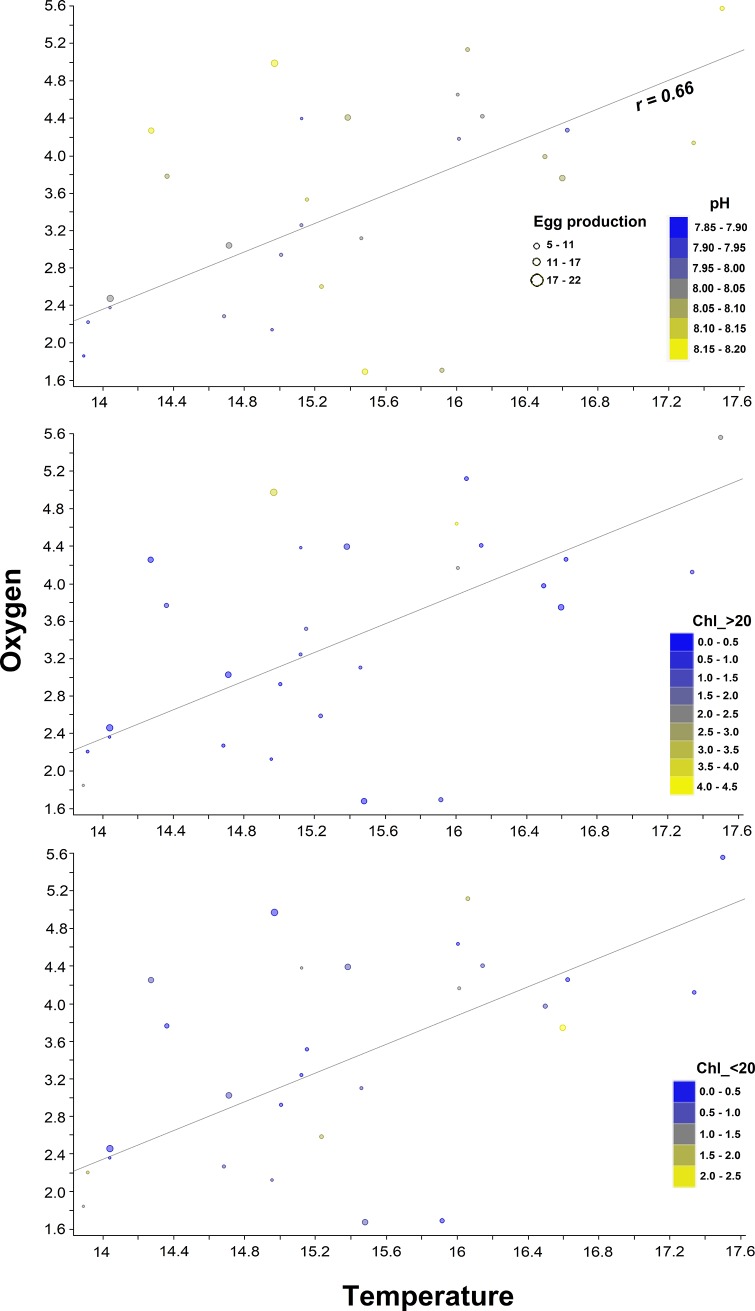
Upwelling modulates copepods reproduction through pH and food variations. (A) Linear (LDA) four-dimensional egg production (EP) ordination as a combined function of temperature, oxygen, pH and Chl. Note how the succession between large (B) and small-sized (C) phytoplankton fractions sustains EP (larger bubbles) despite low temperature, oxygen and pH values.

Coupling between environmental variables was elucidated through a distance map (Fig 5), which was supported by Spearman rank correlations (Table 4). These statistical approaches showed temperature, oxygen, Chl_>20 and pH were significantly and tightly correlated, while other cluster grouped temperature, salinity and Chl_<20. To assess the role of abiotic variables (temperature, salinity, oxygen, pH and Chl) affecting copepod traits (dependent matrix), we first conducted a multivariate linear discriminant (LDA) analysis. The LDA analysis assumes linear combinations of continuous independent variables, which best explain a dependent variable, integrating the techniques of ordination and multiple regressions where quantitative explanatory variables are graphically represented as vectors. Explaining from 49% to 61% of copepod responses variance, this analysis identified temperature and pH as critical factors contributing to modulate morphological and reproductive traits, respectively (Fig 6). A four-dimensional representation of this model, explaining 66% of the EP variability is shown in Fig 7. In addition, we conducted a covariance analysis (ANCOVA) under a generalized linear model assuming an error Type III and considering the cluster node as a categorical predictor. Results of the ANCOVA analysis are shown in Table 5. According to this analysis, only temperature and pH, and pH and body size explained a small proportion of the variability observed in body size (R^2^ = 0.2) and egg size (R^2^ = 0.1), respectively. Furthermore, showing significant correlations with response variables, a model including temperature, oxygen, and pH explained a significant proportion of EP (R^2^ = 0.54) and growth (R^2^ = 0.52) changes.

**Table 4:** Significant (Spearman rank) correlations between environmental factors, which supported the corresponding distance map (see Fig 5).

	Environmental coupling			
	Temperature	pH	Oxygen	Salinity
Temperature				
pH	0.47			
Oxygen	0.73	0.39		
Salinity	0.55		0.30	
Chl_>20	0.52	0.55	0.58	
Chl_<20				0.31

**Table 5:** Results of ANCOVA analysis conducted to analyze variability in copepod traits with regards to oceanographic conditions. Data were normalized, one attribute per value by standard deviation, assuming an error Type III.

		Univariate tests of significance		
Copepod traits	Variability Factor	M.S.	*F*-value	*p*-value
Body size	Temperature	0.14	13.8	0.001
	pH	0.07	7.11	0.01
		*Model R*^*2*^ *= 0*.*2*, *p = 0*.*001*		
Egg production	Temperature	86.5	6.3	0.04
	Oxygen	68.3	10.0	0.002
	pH	1870.5	137	0.001
		*Model R*^*2*^ *= 0*.*54*, *p = 0*.*001*		
Egg size	pH	55.9	8.2	0.005
	Body size	68.3	10.0	0.002
		*Model R*^*2*^ *= 0*.*1*, *p = 0*.*001*		
Growth	pH	1.1	142.0	0.001
	Temperature	0.05	6.2	0.01
		*Model R*^*2*^ *= 0*.*52*, *p = 0*.*001*		

## Discussion

### Nodes of temporal variability

The current study was conducted in a year-round upwelling area exposed frequently to turbulent freshly upwelled waters [37]. Upwelling filaments, involving the surface uplift of Equatorial Sub Surface Waters (ESSW), are characterized by salinity values > 34.6, low temperatures (<17°C), and oxygen and pH levels down to 4.17 mL L^-1^ and 7.7 (pH_@25°C_ units), respectively [1,35,47]. Environmental conditions like these, along with low Chl concentrations (<1 μg L^-1^), especially in the large size fraction (Chl_>20), were observed to occur randomly throughout the study period. These conditions were usually followed by periods of higher values in all environmental parameters. These temporal features, likely representing “upwelling” (node A) and “non-upwelling” (node B) conditions respectively, were captured by both hierarchical agglomerative clustering and MDS tests (Fig 3). However, other factors, such as changes in phytoplankton biomass [48,49], upwelling-driven replacement of water masses [3,5], or Equatorial processes [35,50], can lead to sharp temporal variations in the physical-chemical conditions of the coastal upwelling seascape. In fact, clustering and MDS analyses also identified a third cluster node (node C), which was characterized by the highest temperatures (17°C), oxygen (>4.5 mL L^-1^), salinity (35) and Chl_>20 concentrations (2.5 μg L^-1^), while Chl_<20 and pH levels returned to values observed during “upwelling” conditions (Fig 4). The examination of sampling days grouped into node C (Table 2) and a MDS class density representation (i.e. density of cluster nodes) (S1 File), revealed that while nodes A and B were randomly distributed on MDS planes, “warm-acid” node C was restricted to almost exclusively a particular position (i.e. period time), which corresponded to winter samplings (Table 2). Interestingly, while all oceanographic parameters (temperature, oxygen, salinity) increased (Fig 4), pH decreased until reaching upwelling values. Previous studies in this same upwelling area have shown low oxygen (but not hypoxic, 1.7–4.7 mL L^-1^) and permanently *p*CO_2_-supersaturated (> 800 μatm CO_2_) and low pH (< 7.9 pH units) ESSW can reach the surface despite the ENSO effects on physical-chemical properties of the water column [1,35].

### Environmental and biological coupling

The distance map among abiotic parameters evidenced the transition of environmental conditions in the water column from nodes A to B, or the transition from fresh to old upwelled waters. According to the distance map (Fig 5), freshly upwelled waters characterized by low temperature and oxygen, first impacted phytoplankton biomass, among which the large size fraction (Chl_>20) seemed to react first. Thermal impact likely affected the suitability of the aquatic environment for phytoplankton growth and production under non-limiting light conditions [36]. This is because Chl concentrations relatively lower than values generally reported inside the two-nearby embayment under the influence of upwelling “shadows” (> 8 μg L^-1^ [36]) prevailed in the upwelling site. Despite this, phytoplankton biomass seemed to partially buffering the surface acidification by sub surface waters, given the significant correlations between Chl, pH and oxygen (Table 4).

With regards to our hypothesis that copepod traits were unaffected by upwelling pH-changes, multivariate LDA analyses revealed each copepod trait had a specific combination of controlling factors, among which, seawater pH played a major role (Fig 6). These findings were supported by results of an ANCOVA analysis, which though in most cases reduced both the power of the fit and the number of significant explanatory variables (Table 5), it still maintained temperature, oxygen and pH as significant factors controlling egg production (R^2^ = 0.54) and growth (R^2^ = 0.52). It seems that upwelling, through low temperature, oxygen and also through low pH, can affect the fitness of these planktonic grazers. In fact, temperature and oxygen have been recognized as critical factors controlling body size [37,51,52] and physiological rates of pelagic copepods, respectively [53,54]. Egg production is a particularly sensitive physiological trait, since this is considered an approach to estimate population growth or copepod secondary production [44,55]. Since once adult, small-sized species like *A*. *tonsa* invest almost all their energy into egg production [56,57], a tight relationship is thus expected between egg production and the immediate environmental experience of adult females [45,58]. Previous studies have found that *A*. *tonsa* and other small-sized copepod species as well, display relatively low but temporally constant egg production rates in these upwelling regions, which frequently are not related or limited by ambient food or temperature [13,14,15,17,59]. This current multiparametric study indicated that in combination with temperature and oxygen, pH could have a significant impact on morphometric, and especially on reproductive traits of *A*. *tonsa*. Either buffering upwelling pH-reductions or alleviating elevated energetic demands under stressful conditions observed during intense upwelling [60,61], subtle increases of phytoplankton biomass seems to sustain copepod reproduction outputs despite the frequent upwelling episodes affecting the study area (Fig 7). Importantly, Chl concentration is only an index of food resources for *A*. *tonsa*, which tend to be highly efficient preying on heterotrophic components that are temporally stable in sub-tropical upwelling ecosystems of HCS [59].

**Fig 8:**
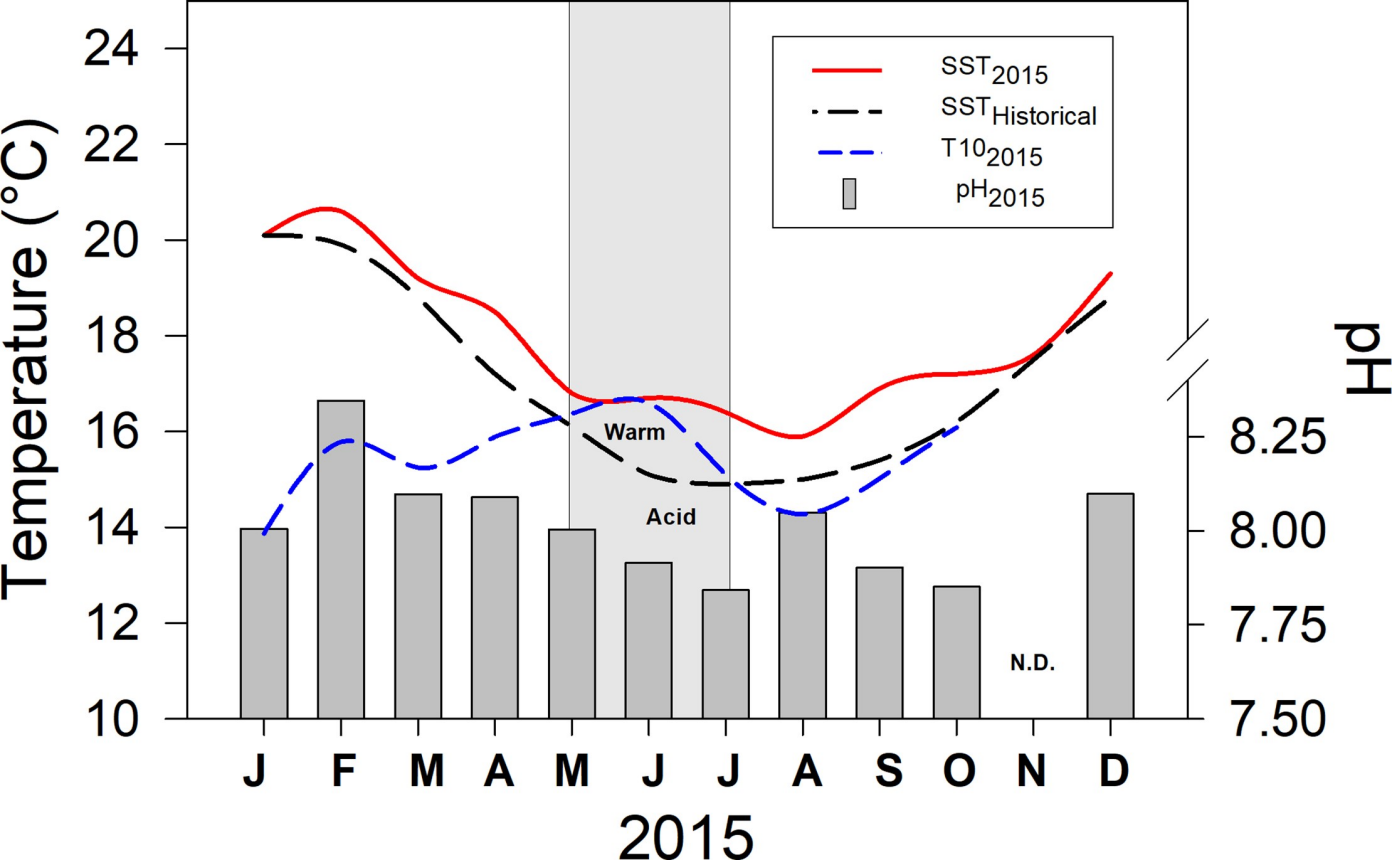
Historical oceanography vs. “El Niño” 2015. Sea surface temperature during 2015 (SST_2015_) and historical data (1981–2010, SST_Historical_) are plotted along with temperature (T10) and pH measured in this study.

Warm conditions observed in cluster node C configure positive deviations from local climatology [62], projecting oceanographic symptoms likely associated with El Niño 2015 [63]. Monthly means of sea-surface temperature (SST) observed during the year 2015 as well as historical SST values (1981–2010) collected in Mejillones Bay (Fig 1), were taken from the database of the Hydrographic Service of the Chilean Navy (SHOA) and plotted along with temperature (T10) and pH observed at 10 m depth during this study (Fig 8). Sustained increase in T10 since April suggests the onset of the event but also might evidence the deepening of the thermocline with the arrival of the Kelvin waves [64]. Likely decoupling the capacity of phytoplankton biomass for buffering upwelling low pH/high CO_2_ conditions, this event was followed by a sustained pH decrease and thus, the “warm-acid” period lasted roughly two months and lead to a significant reduction in most copepod traits. Focusing on growth rate (which in its calculation involves the body size, egg production and egg size), current findings seems to be in disagreement with previous studies suggesting copepods growth increased during warming El Niño conditions [37,64]. Whereas updated pH measurements [40] have been only recently coupled to routine biological oceanographic studies in HCS [7], previous studies considered a large-sized species and were often conducted in upwelling shadows [37,51,52]. Large-sized *Calanus chilensis* perform deeper and colder ontogenic migrations than *A*. *tonsa* and thus, thermal changes due to ENSO but also to upwelling fronts can trigger a sharped impact on its enzyme activity and metabolism [37,52]. Furthermore, since low-pH, freshly upwelled waters in open areas experience drastic physical-chemical modifications after entering into the bay and give place to upwelling shadows, current findings with a neritic species under “warm-acid” conditions provided a valuable point of comparison to understand variations in secondary production under variable upwelling conditions.

### Ecology underpinning copepod responses to future upwelling scenarios

The capacity of marine organisms to cope with environmental perturbations depends on how variable the species’ habitat is [7,27]. Similar than most of the small-medium size copepod species that dominate the coastal assemblage in the HCS and other EBUS, the vertical expansion of the trophic niche of *A*. *tonsa* is restricted by a shallow oxygen minimum zone, which is also characterized by corrosive (e.g. low pH) conditions [17,31,32,35]. Changes in the vertical expansion of trophic niche as a consequence either of phenotypic plasticity within *A*. *tonsa* populations or specie-specific variations, can emerge as a key mechanism underpinning responses of planktonic grazers to cope with pH-perturbations associated with climate change [29]. Either buffering pH reductions or alleviating energetic demands under stressing upwelling conditions, phytoplankton biomass seems to modulate biological impacts due to upwelling intensification. Nearby bays under the influence of upwelling shadows and leading to an improved phytoplankton and copepod fitness [36,37], might thus represent climate change refuges upon future more intense upwelling conditions expected to occur in EBUS [9].

## Supporting information

S1 File Class density of clustering nodes. Two-dimensional representations of di-similitude matrices showing cluster C interrupting the random transition from nodes A to B.
(PNG)

S2 File Grammar editor name. (XLS)

S3 File Data set. (DOCX)
