# Supplementary figures and images for: Upwelling modulation of functional traits of a dominant planktonic grazer during “warm-acid” El Niño 2015 in a year-round upwelling area of Humboldt Current
Source: PLoS One. 2019 Jan 14;14(1):e0209823. doi: 10.1371/journal.pone.0209823 (PMC6331177; doi:10.1371/journal.pone.0209823)

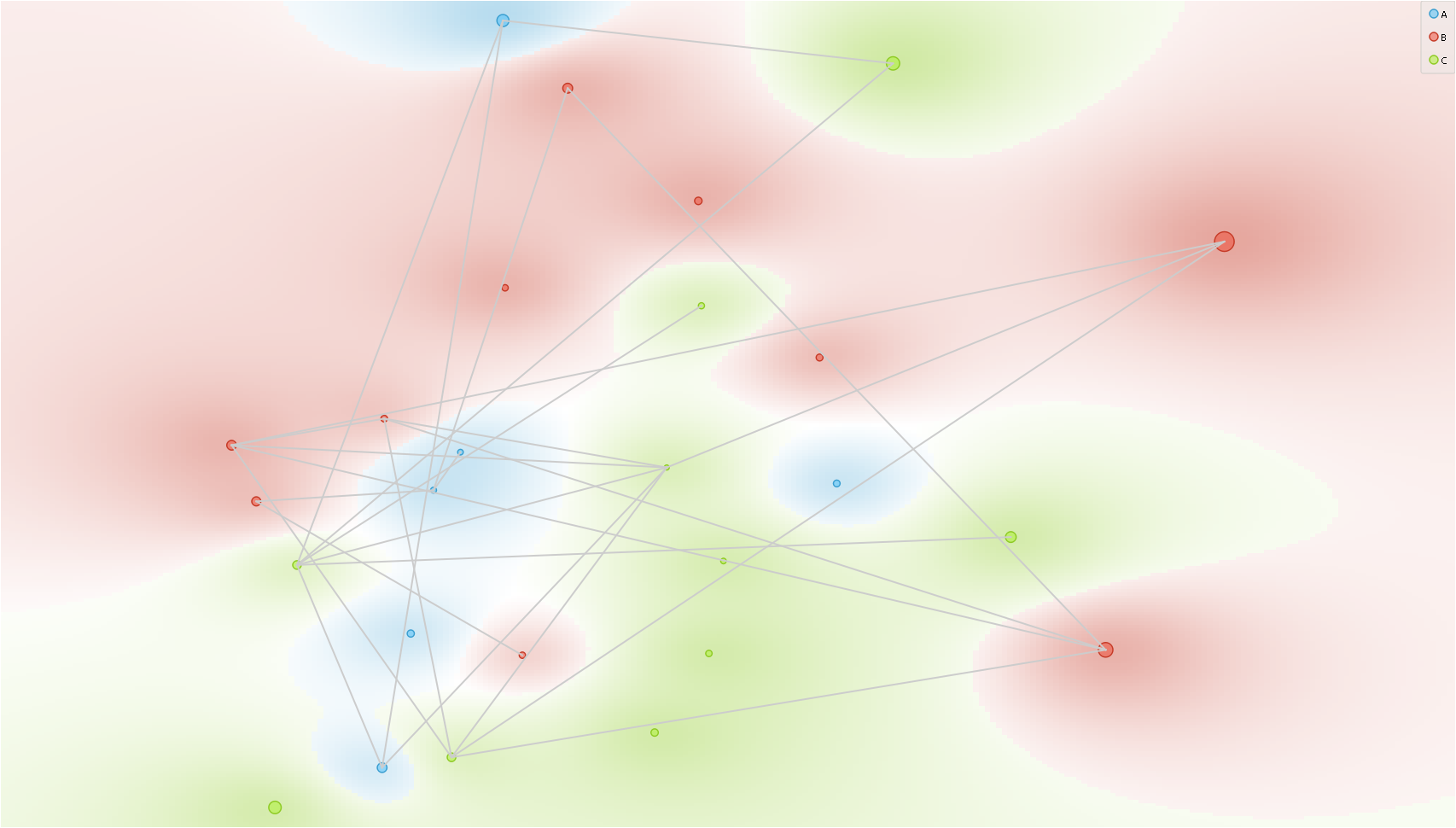

Supplement: S1 File — Two-dimensional representations of di-similitude matrices showing cluster C interrupting the random transition from nodes A to B. (PNG) [file pone.0209823.s001.png]
