# Supplementary material for: Upwelling modulation of functional traits of a dominant planktonic grazer during “warm-acid” El Niño 2015 in a year-round upwelling area of Humboldt Current
Source: PLoS One. 2019 Jan 14;14(1):e0209823. doi: 10.1371/journal.pone.0209823 (PMC6331177; doi:10.1371/journal.pone.0209823)
Supplement: S3 File — (DOCX) [file pone.0209823.s003.docx]

Grammar editor

Ms. Charlie M. Zeise
